# High-Efficiency Cryopreservation of Silver Pomfret Sperm: Protocol Development and Cryodamage Assessment

**DOI:** 10.3390/ani15243602

**Published:** 2025-12-15

**Authors:** Man Zhang, Yijun Jiang, Yubei Qiu, Zukang Feng, Xianglong Chen, Chongyang Wang, Yuanbo Li, Qinqin Dai, Jiabao Hu, Xiaojun Yan, Yajun Wang

**Affiliations:** 1 Key Laboratory of Applied Marine Biotechnology, Ningbo University, Ministry of Education, Ningbo 315211, China; 2 Key Laboratory of Marine Biotechnology of Zhejiang Province, Ningbo University, Ningbo 315211, China; 3 College of Marine Sciences, Ningbo University, Ningbo 315211, China

**Keywords:** *Pampus argenteus*, cryopreservation, sperm motility, CASA, ultrastructure

## Abstract

The silver pomfret (*Pampus argenteus*), widely distributed across the Indo-West Pacific and prevalent in China’s coastal waters, has experienced significant resource decline due to anthropogenic impacts such as habitat alteration and overfishing, which disrupt its natural reproduction and growth. Cryopreservation technology overcomes spatiotemporal constraints by enabling the long-term storage of high-quality sperm for future use. This study optimized cryopreservation protocols for silver pomfret sperm, evaluation key parameters including extenders, cryoprotectants, dilution ratios, cooling heights, and thawing temperatures. Sperm quality was assessed post thaw via enzyme activity assays and electron microscopy. Results demonstrated that modified plaice Ringer solution (MPRS) extender yielded the highest post-thaw motility (95.98 ± 1.59)%. The optimal cryopreservation conditions for silver pomfret sperm were established as follows: MPRS diluent, 20% EG, a 1:6 dilution ratio, a 7 cm cooling height, and a 28 °C thawing temperature. This protocol yielded post-thaw sperm with motility and motion parameters most closely resembling those of fresh sperm. Ultrastructural observations and enzyme activity assays, however, confirmed that cryopreservation induced sublethal damage, including significant reduction in ATPase activity, as well as structural anomalies such as head deformation, membrane damage, and organelle disarray. This work establishes a foundational cryopreservation protocol, providing critical tools for conserving the genetic resources of this declining species and supporting sustainable aquaculture and wild population restoration efforts.

## Simple Summary

Silver pomfret (*Pampus argenteus*), widely distributed in the Indo-West Pacific, has declined due to human activities (habitat alteration, overfishing) harming its reproduction. Cryopreservation enables long-term high-quality sperm storage, so this study optimized sperm cryopreservation protocol by testing extenders, cryoprotectants, etc., and assessing post-thaw quality via enzyme assays and electron microscopy. Results: The optimal cryopreservation conditions for silver pomfret sperm were established as follows: MPRS diluent, 20% EG, a 1:6 dilution ratio, a 7 cm cooling height, and a 28 °C thawing temperature. Cryopreservation reduced ATPase but increased glutathione reductase activity and caused cryodamage. This protocol aids silver pomfret genetic resource conservation, sustainable aquaculture and wild population restoration.

## 1. Introduction

The silver pomfret (*Pampus argenteus*) belongs to the order Perciformes, family Stromateidae, and genus *Pampus*. It is widely distributed across the Indo-West Pacific region and present in all of China’s coastal areas, holding significant commercial value as a marine fish species [1]. However, since the 1990s, overfishing and habitat degradation have led to a severe decline in its wild populations [2]. To meet market demand and reduce fishing pressure on natural stocks, the development of artificial breeding techniques for this species has become increasingly urgent. Considerable progress has been made in understanding its reproductive biology [3,4], nutritional requirements [5], larval rearing techniques [6], and reproductive physiology [7]. Despite these advances, research on sperm cryopreservation—a crucial technology for germplasm repository establishment and assisted reproduction—remains underdeveloped for this species.

Successful cryopreservation of fish sperm depends on the careful optimization of multiple technical parameters, including suitable extender composition, effective yet safe cryoprotectants and their optimal concentrations, appropriate dilution ratios, and controlled cooling protocols [8,9]. Extensive studies on phylogenetically related species and other economically important fish provide valuable references. For instance, a combination of 10% DMSO and 300 mM glucose has been shown to be an effective cryoprotective solution for sperm of the red-spotted grouper, *Epinephelus akaara* [10]. In Basa catfish (*Pangasius bocourti*), a dilution ratio of 1:3 yielded the best cryopreservation outcomes [11]. Similarly, for the brown-marbled grouper (*Epinephelus fuscoguttatus*), thawing in a 30–40 °C water bath was reported to yield superior sperm motility recovery [12], while for the seven-band grouper (*Epinephelus septemfasciatus*), post-thaw activation rates showed no significant differences across freezing heights ranging from 2.5 to 10 cm above the liquid nitrogen surface [13]. These findings collectively underscore the strong species-specificity of successful cryopreservation protocols.

Although cryopreservation technology continues to advance, cryodamage remains unavoidable to some extent [14]. Therefore, a comprehensive and multi-faceted evaluation of post-thaw sperm quality is essential. Beyond assessing motility parameters (such as motility rate and kinematic characteristics), measuring enzymatic activities—for instance, ATP content as an indicator of energy metabolism and lactate dehydrogenase as a marker of cellular integrity—can provide insights into the physiological status and extent of cryo-injury [15]. Furthermore, genetic integrity can be assessed through single-cell gel electrophoresis (comet assay) [16,17], while cell membrane integrity is commonly determined using microscopic evaluation of sperm staining [18,19]. At the structural level, ultrastructural examination using electron microscopy enables direct assessment of organelle integrity (mitochondria and flagella). This approach has been validated as an effective means of evaluating cryodamage in sperm, as demonstrated in studies on species such as the yellow croaker (*Larimichthys polyactis*) [20], olive flounder (*Paralichthys olivaceus*) [21], and striped bass (*Morone saxatilis*) [22].

In summary, while sperm cryopreservation has been successfully applied in various fish species, a standardized and efficient protocol for silver pomfret has not yet been established, and the physiological and structural changes in its sperm during cryopreservation–thawing remain poorly understood. Therefore, this study aims to develop an effective cryopreservation procedure for silver pomfret sperm by systematically screening key parameters, including extender, cryoprotectant concentration, dilution ratio, freezing height, and thawing temperature. The post-thaw sperm quality will be comprehensively evaluated using computer-assisted sperm analysis (CASA), enzymatic activity assays, and transmission electron microscopy. The findings will provide core technical support for establishing a silver pomfret germplasm resource bank, which is vital for the conservation and sustainable utilization of this valuable marine resource.

## 2. Materials and Methods

### 2.1. Fish Source

The silver pomfret used in this experiment were obtained from Xiangshan Harbor Aquaculture and Larva Company Ltd. (Ningbo, Zhejiang Province, China). Sexually mature one-year-old male fish (average body weight: 88.96 ± 9.37 g) were reared in a 40-ton circular cement tank with a daily water exchange rate of 100%. The water quality parameters were maintained as follows: dissolved oxygen = 8.95 ± 0.2 mg/L, salinity = 23 ± 1‰, pH = 8.16 ± 0.3, and a photoperiod of 12 h light/12 h dark. The fish were fed four times daily with Haitong No. 6 floating pellet feed (crude protein ≥ 55%, crude fat ≥ 8%) at a daily feeding rate of 3% of their body weight. Prior to the experiment, the fish were acclimated for two weeks under the same conditions, and feeding was withheld on the day before sampling.

Since artificial spawning induction techniques are not yet standardized or widely established for silver pomfret, gametes were collected manually in this study. To ensure sufficient semen volume for subsequent experimental procedures, milt was collected from a total of 36 male individuals. The mature males each provided 0.3–1 mL of semen. A total of 36 males yielded approximately 25 mL, providing a sufficient volume for the experimental requirements. Semen from every six fish was pooled and stored for subsequent experiments.

### 2.2. Sperm Collection

Sexually mature males were netted and anesthetized using buffered MS-222 (tricaine methanesulfonate). The abdominal region and urogenital opening were gently blotted with sterile absorbent paper. Then, sperm was expressed by applying gentle abdominal pressure. The initial portion (potentially contaminated with urine or feces) was discarded. After re-blotting the urogenital area, sperm was aspirated using a sterile micropipette. A total of 36 males yielded approximately 25 mL, providing a sufficient volume for the experimental requirements. Semen from every six fish was pooled and stored on crushed ice (at approximately 4 °C) and processed within 1 h. All procedures were performed wearing sterile gloves with instruments disinfected in 75% ethanol and air-dried to prevent sample contamination and premature sperm activation. Sperm quality was preliminarily assessed under light microscopy (NI-SS, Nikon, Tokyo, Japan). Only samples meeting high-quality standards (≥90% post-collection motility without contamination or abnormal activation) were used. The evaluations were also performed after activating the spermatozoa with seawater.

### 2.3. Extender Preparation

Five extenders (MPRS, Cortland, HBSS, Hank’s, and Ringer’s solutions) were prepared according to Table 1. All solutions were stored at 4 °C for pre-equilibration before use.

### 2.4. Evaluation of Extender Types

Five extenders (HBSS, MPRS, Cortland, Hank’s, and Ringer’s solutions) were mixed with 20% Ethylene Glycol [23] based on preliminary data and literature on related species, generating five cryopreservation solutions. These solutions were pre-cooled at 4 °C. Throughout the procedure, the liquid nitrogen level was maintained at 30 cm by periodic replenishment. Sperm was diluted 1:6 (*v*/*v*) with each cryopreservation solution, loaded into 0.25 mL French straws, and equilibrated at 4 °C for 10–15 min. Liquid nitrogen was poured into a 50 × 40 × 40 cm polystyrene foam box to a depth of 30 cm. A 7 cm-high foam platform—featuring an open bottom and grooves at both ends designed to secure cryopreservation straws—was then floated on the liquid nitrogen surface. Straws were then vapor-cooled on the platform for 5 min before rapid immersion in liquid nitrogen for storage. After 24 h, samples were thawed in a 28 °C water bath for 10 s. Post-thaw sperm motility was assessed using computer-assisted sperm analysis (CASA) [23] after activating 1 µL sperm with 5 µL sterile artificial seawater. A pooled milt sample collected from six fish was used in this experiment, with each treatment group assayed in triplicate.

### 2.5. Evaluation of Permeable Cryoprotectants

MPRS extender was supplemented with DMSO, EG, PG, MeOH, or Gly at final concentrations of 5%, 10%, 15%, or 20%. After pre-cooling at 4 °C, sperm was diluted 1:6 (*v*/*v*) with each of the twenty cryopreservation solutions. Subsequent procedures (loading, cooling, storage, thawing, and motility analysis) followed Section 2.4. A pooled milt sample collected from six fish was used in this experiment, with each treatment group assayed in triplicate.

### 2.6. Evaluation for Dilution Ratio, Cooling Height, and Thawing Temperature

Sperm was diluted with MPRS extender containing 20% EG at ratios of 1:1, 1:3, 1:6, and 1:9 (*v*/*v*).

Samples prepared under optimal conditions (MPRS + 20% EG, 1:6 dilution) were vapor-cooled at heights of 3 cm (−161 ± 5.2 °C), 5 cm (−139 ± 4.3 °C), 7 cm (−122 ± 3.8 °C), and 9 cm (−101 ± 4.7 °C) above liquid nitrogen for 5 min before cryostorage. Specifically, “cooling height” and “freezing height” as “the height above the liquid nitrogen surface”.

Cryopreserved sperm were thawed in water baths at 18 °C, 23 °C, 28 °C, or 33 °C for 10 s. Motility analysis was conducted as in Section 2.4. A pooled milt sample collected from six fish was used in this experiment, with each treatment group assayed in triplicate.

### 2.7. Sperm Kinetic Analysis Using CASA

Sperm motility parameters were quantitatively assessed using a CASA system (CASAS-MX7, Tsinghua Tongfang, Beijing, China). Briefly, a 3 mL aliquot of thawed sperm was placed on a pre-warmed (4 °C) standard counting chamber (10 μm depth slide, Tsinghua Tongfang, Beijing, China). The sample was immediately activated with 30 μL of sterilized seawater at room temperature, and mixing was achieved by gently drawing the solution in and out of a pipette tip. The slide was then placed on the thermostatically controlled stage of the microscope (NI-SS, Nikon, Tokyo, Japan) at room temperature. For each sample, a minimum of ten random fields were captured (SSC-G108, SONY, Minato, Japan). The system settings were configured as follows: frame rate (1920 × 1080); 30 of frames captured. Spermatozoa were considered motile when their average path velocity (VAP) exceeded 10 μm/s.

The following kinematic parameters were measured and analyzed:

Total Motility (%): The percentage of all spermatozoa exhibiting any form of movement.

Progressive Motility (%): The percentage of spermatozoa moving in a forward direction with a straightness (STR) value exceeding 80%.

Curvilinear Velocity (VCL, μm/s): The time-average velocity of a sperm head along its actual curvilinear path.

Average Path Velocity (VAP, μm/s): The time-average velocity of a sperm head along its spatial average path.

Straight-Line Velocity (VSL, μm/s): The time-average velocity of a sperm head along the straight line from its first to its last position.

The selected parameters, which are widely reported in fish sperm cryopreservation studies, are linked to sperm motility and fertilizing ability.

### 2.8. Effect of Cryopreservation on Sperm Enzyme Activity

ATP content (A095-1-1, Jiancheng Bioengineering, Nanjing, China), glutathione reductase (A062-1-1, Jiancheng Bioengineering, Nanjing, China) and lactate dehydrogenase (A020-2-1, Jiancheng Bioengineering, Nanjing, China) activities were measured in fresh and cryopreserved sperm using commercial kits. Prior to assay, semen samples were processed to extract intracellular components. Specifically, 100 µL volume of semen was mixed with 900 µL of the kit-provided extraction buffer. The mixture was thoroughly homogenized by vortexing for 30 s, followed by ultrasonic disruption on ice (3 cycles of 5 s pulses at 40% amplitude with 10 s intervals) to ensure complete cell lysis and release of intracellular ATP and enzymes. The lysate was then centrifuged at 10,000× *g* for 10 min at 4 °C to remove cellular debris and membranes. The resulting clear supernatant was carefully collected and used immediately for the subsequent assays. The cryopreservation of sperm followed the optimal protocol (MPRS + 20% EG, 1:6 dilution rates, at 7 cm height) identified in the previous experiments. Assays were performed according to manufacturer specifications regarding reaction systems, temperatures, and incubation times. The protein concentration of the supernatant was determined using a compatible protein assay kit (BCA method) to normalize enzyme activity values, where required by the assay principle. A pooled milt sample collected from six fish was used in this experiment, with each treatment group assayed in triplicate.

### 2.9. Ultrastructural Analysis of Cryopreserved Sperm

Transmission Electron Microscopy (TEM): Fixed and washed samples (cryopreserved sperm using the optimal protocol (MPRS + 20% EG, 1:6 dilution rates, at 7 cm height) identified in previous experiments, *n* = 6) were dehydrated, embedded in Spurr resin, and polymerized at 70 °C for 24 h. Ultrathin sections (70 nm) were stained with 2% uranyl acetate and lead citrate before observation using a Hitachi HT-7800 TEM (Chiyoda, Japan).

### 2.10. Statistical Analysis

Data were processed in Excel 2021 (outlier screening, normalization) and analyzed using SPSS 27.0. Data normality was verified using the Shapiro–Wilk test. For normally distributed data, one-way ANOVA was applied, followed by Tukey’s HSD post hoc test for significant differences. Results are presented as mean ± SEM and visualized using GraphPad Prism 8.2.1.

## 3. Results

### 3.1. Evaluation of Extenders for Silver Pomfret Sperm Cryopreservation

**Figure 1:**
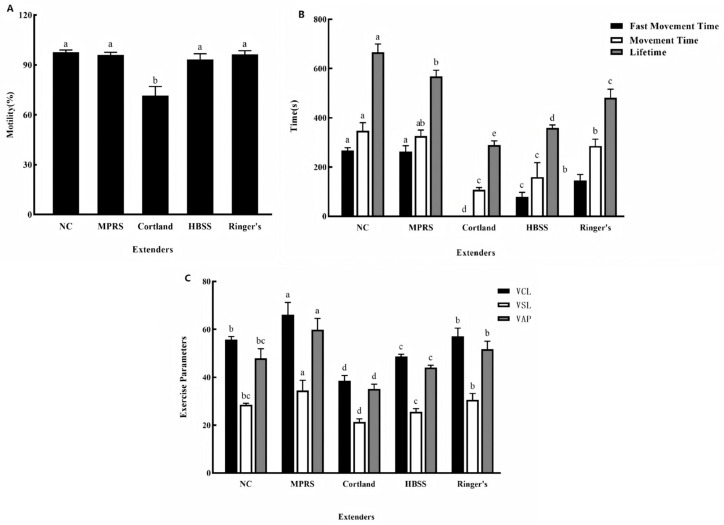
(**A**) Effects of different diluents on the motility of silver pomfret sperm during cryopreservation. NC: Fresh sperm. (**B**) Effects of different extenders in cryopreservation on fast movement time, movement time, and lifetime of silver pomfret sperm. (**C**) Effects of different extenders on sperm kinetic parameters during cryopreservation. The number of biological replicates (males, *n* = 6); one-way ANOVA; bars represent mean ± SD; different letters indicate significant differences.

As shown in Figure 1A, sperm preserved in Cortland solution exhibited a significantly lower motility rate compared to the other groups (*p* < 0.05).

The kinetic parameters of fresh sperm were superior to all cryopreserved groups, displaying the longest fast movement time (665.67 s), movement time (347 s), and lifetime (267.33 s) (Figure 1B). Among the cryopreserved groups, sperm in MPRS most closely mimicked fresh sperm, with no significant differences in fast movement time and movement time, despite a significant decrease in lifetime (568 s; *p* < 0.05). Sperm preserved in Cortland, HBSS and Ringer’s solutions showed significantly impaired performance across all parameters (*p* < 0.05).

As shown in Figure 1C, sperm cryopreserved in MPRS achieved the highest values for the kinematic parameters VCL (66.12 µm/s), VSL (34.54 µm/s), and VAP (59.8 µm/s), which were even superior to those observed in fresh sperm (*p* < 0.05). Sperm preserved in Ringer’s solution exhibited no significant differences in these motility parameters compared to fresh sperm. For sperm cryopreserved in HBSS solution, VSL and VAP showed no significant difference from fresh sperm, whereas VCL was significantly lower. In contrast, sperm preserved in Cortland solution yielded the lowest values for all recorded motility parameters, which were significantly inferior to all other groups.

### 3.2. Effects of Different Cryoprotectants on the Preservation Outcomes of Silver Pomfret Sperm

**Figure 2:**
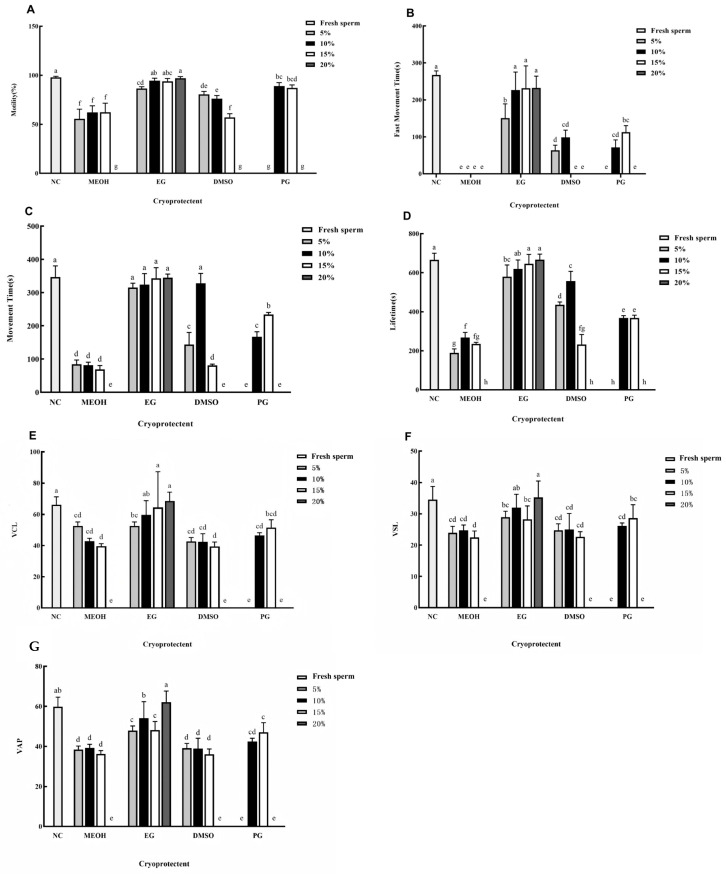
Effects of different cryoprotectants and their concentrations on the preservation of silver pomfret sperm. (**A**) Sperm motility. (**B**) Fast movement time. (**C**) Movement time. (**D**) Lifetime. (**E**) VCL. (**F**) VSL. (**G**) VAP. Note: NC. Fresh sperm; MEOH. Methanol; EG. Ethylene glycol; DMSO. Dimethyl sulfoxide; PG. Propylene glycol; 5–20% indicates the final concentration of the cryoprotectant; MPRS extender mixed with 20% EG. The number of biological replicates (males, *n* = 6); one-way ANOVA; bars represent mean ± SD; different letters indicate significant differences. The sperm in the glycerol group all died.

Figure 2 illustrates the effects of different types and concentrations of cryoprotectants on the cryopreservation of silver pomfret sperm. As shown in Figure 2A, the fresh sperm group exhibited the highest motility (97.77%). No significant differences in post-thaw motility were observed between the fresh sperm and the groups treated with 10%, 15%, and 20% EG. In contrast, all other cryoprotectant groups showed significantly lower motility compared to fresh sperm.

In terms of fast movement time (Figure 2B), fresh sperm recorded 267.33 ± 11.02 s. The groups cryopreserved with 10%, 15%, and 20% EG showed comparable results to fresh sperm, with no statistically significant differences, whereas all other groups were significantly lower. Notably, sperm treated with different concentrations of MEOH exhibited a fast movement time of zero.

Sperm movement time is presented in Figure 2C. Compared to fresh sperm, no significant differences were detected in groups treated with various concentrations of EG or with 10% DMSO. All other groups demonstrated significantly shorter movement time.

As depicted in Figure 2D, sperm lifetime in the 10%, 15%, and 20% EG groups was not statistically different from that of fresh sperm, while all other cryoprotectant groups resulted in significantly reduced lifetime.

Regarding VCL (Figure 2E), again, the 10%, 15%, and 20% EG groups showed no significant difference from fresh sperm, unlike all other groups which showed significantly lower VCL.

For straight-line VSL (Figure 2F), no significant difference from fresh sperm was observed in the 10% and 20% EG groups. All other treatments led to significantly reduced VSL.

Finally, the VAP (Figure 2G) was not significantly different from that of fresh sperm only in the 20% EG group. All other cryoprotectant groups yielded significantly lower VAP values.

Collectively, based on the comprehensive parameters evaluated, 20% EG was identified as the optimal cryoprotectant for sperm cryopreservation.

### 3.3. Effects of Different Sperm Dilution Ratios on Cryopreservation Outcomes

**Figure 3:**
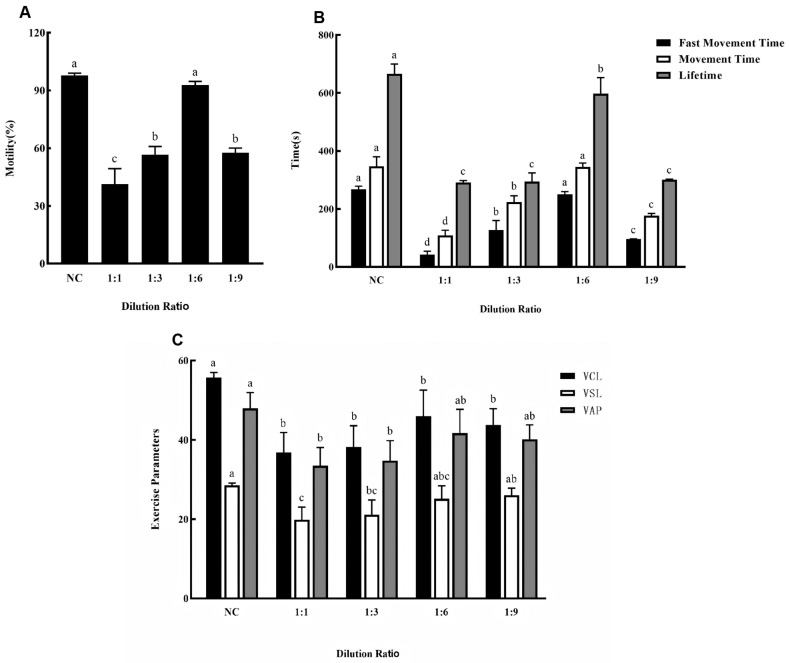
(**A**) Effects of different dilution ratios on the motility of silver pomfret sperm during cryopreservation. NC: Fresh sperm. (**B**) Effects of different dilution ratios in cryopreservation on fast movement time, movement time and lifetime of silver pomfret sperm (the black bars in the figure show average fast movement time; the white bars show average movement time; the grey bars show average lifetime). (**C**) Effects of different extenders on sperm kinetic parameters during cryopreservation. The number of biological replicates (males, *n* = 6); one-way ANOVA; bars represent mean ± SD; different letters indicate significant differences.

Based on our previous findings, MPRS was selected as the extender and 20% EG as the cryoprotectant for sperm cryopreservation. The effect of different sperm-to-extender dilution ratios on post-thaw quality is shown in Figure 3.

Figure 3A illustrates the sperm motility rate. The 1:6 dilution group showed a motility rate most comparable to that of fresh sperm, with no significant difference observed. In contrast, the other three dilution groups exhibited significantly lower motility rates compared to fresh sperm.

Similarly, as shown in Figure 3B, with the exception of a significantly reduced lifetime, the 1:6 dilution group showed no significant differences in fast movement time or movement time compared to fresh sperm. The other three dilution groups, however, demonstrated significant reductions in fast movement time, movement time, and lifetime relative to fresh sperm.

Sperm kinematic parameter analysis (Figure 3C) revealed that both the 1:6 and 1:9 dilution groups maintained VSL and VAP values that were not significantly different from those of fresh sperm, although their VCL values were significantly lower. In comparison, the 1:1 and 1:3 dilution groups showed significantly lower values across all measured kinematic parameters relative to fresh sperm.

Based on the above results, the 1:6 sperm-to-extender dilution ratio demonstrated the minimal detrimental impact on sperm quality during cryopreservation.

### 3.4. Effects of Different Cooling Height on Cryopreservation Outcomes

**Figure 4:**
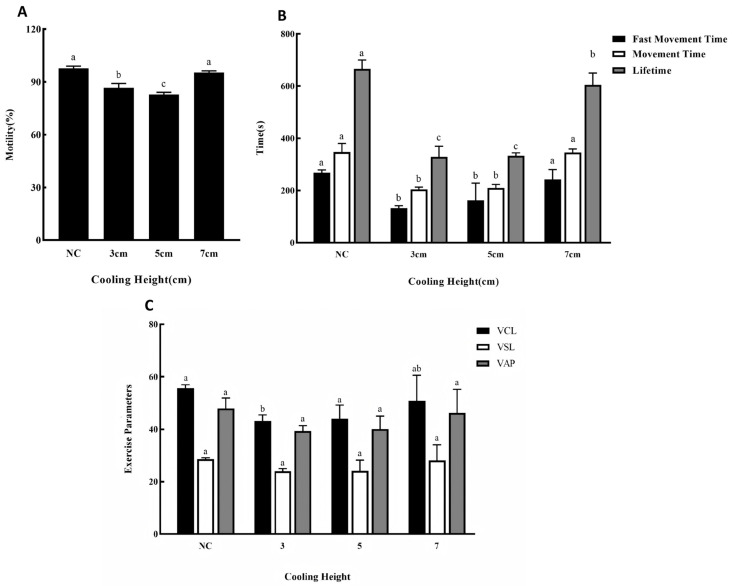
(**A**) Effects of different cooling height on the motility of silver pomfret sperm during cryopreservation. NC: Fresh sperm. (**B**) Effects of different cooling height in cryopreservation on fast movement time, movement time, and lifetime of silver pomfret sperm (the black bars in the figure show average fast movement time; the white bars show average movement time; the grey bars show average lifetime). (**C**) Effects of different extenders on sperm kinetic parameters during cryopreservation. The number of biological replicates (males, *n* = 6); one-way ANOVA; bars represent mean ± SD; different letters indicate significant differences.

Figure 4 illustrates the effect of cooling height on sperm cryopreservation outcomes. The 9 cm group is not shown in Figure 4 because almost no live sperm were observed in the field of view after revival and activation. As seen in Figure 4A, the sperm motility rate in the 7 cm group was not significantly different from that of the fresh control. In contrast, the motility rates in both the 3 cm and 5 cm groups were significantly lower. Figure 4B shows that, except for a significant reduction in sperm lifetime, the fast movement time and movement time in the 7 cm group were comparable to the fresh control. The 3 cm and 5 cm groups, however, exhibited significantly shorter fast movement time, movement time, and lifetime. According to Figure 4C, the kinetic parameters (VCL, VSL, VAP) were less affected by the cooling height. The values for the 5 cm and 7 cm groups showed no significant differences from the fresh control. Although the VCL in the 3 cm group was significantly lower, its VSL and VAP remained similar to the control. In summary, based on the comprehensive evaluation of these indicators, a cooling height of 7 cm yielded the most effective cryopreservation result for silver pomfret sperm.

### 3.5. Effects of Different Thawing Temperatures on Cryopreservation Outcomes

**Figure 5:**
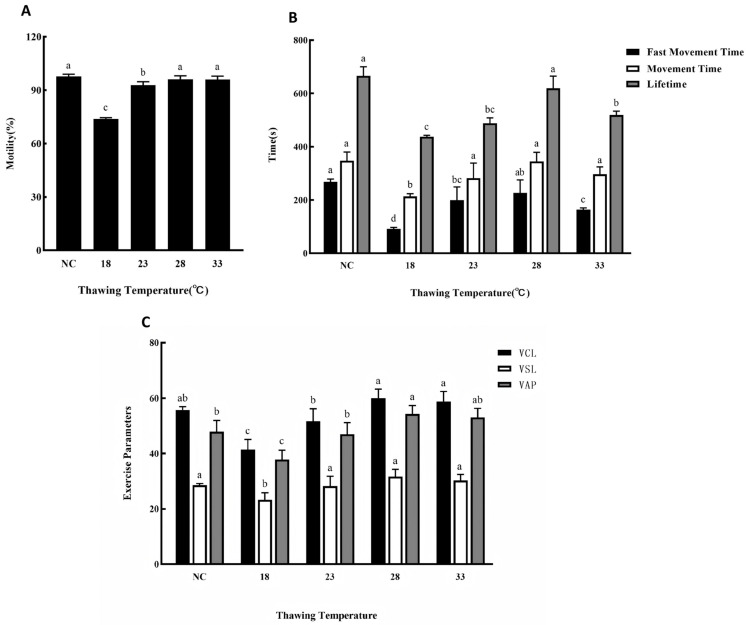
(**A**) Effects of different thawing temperature on the motility of silver pomfret sperm during cryopreservation. NC: Fresh sperm. (**B**) Effects of different thawing temperature in cryopreservation on fast movement time, movement time and lifetime of silver pomfret sperm (the black bars in the figure show average fast movement time; the white bars show average movement time; the grey bars show average lifetime). (**C**) Effects of different extenders on sperm kinetic parameters during cryopreservation. The number of biological replicates (males, *n* = 6); one-way ANOVA; bars represent mean ± SD; different letters indicate significant differences.

As shown in Figure 5, sperm cryopreserved using MPRS as the extender and 20% EG as the cryoprotectant were thawed at different water bath temperatures: 18 °C, 23 °C, 28 °C, and 33 °C. The results indicated that sperm motility showed no significant difference from the fresh control when thawed at 28 °C and 33 °C, while it was significantly lower at 18 °C and 23 °C (Figure 5A). As shown in Figure 5B, the fast movement time, movement time, and lifetime at a thawing temperature of 28 °C were not significantly different from those of fresh sperm. At 23 °C and 33 °C, the movement time was comparable to the control, but both the fast movement time and lifetime were significantly reduced. All three motility parameters were significantly lower at 18 °C than in the fresh control. The sperm kinematic parameters under different thawing temperatures are presented in Figure 5C. The VCL, VSL, and VAP values at 28 °C and 33 °C showed no statistical differences from the fresh control. At 23 °C, only the VSL was not significantly different from the control, whereas both VCL and VAP were significantly lower. In contrast, all three kinematic parameters were significantly lower at 18 °C. In summary, a thawing temperature of 28 °C had a minimal impact on post-thaw sperm quality.

### 3.6. Effect of Ultra-Low-Temperature Cryopreservation on Enzyme Activity of Silver Pomfret Sperm

**Figure 6:**
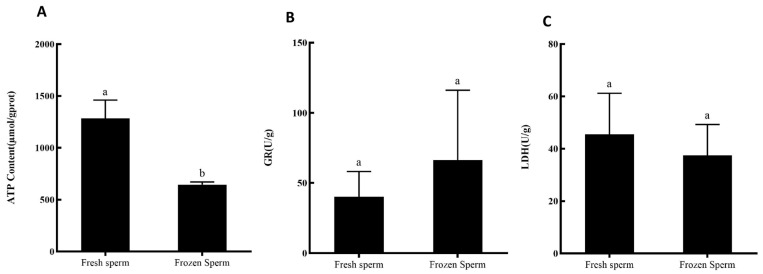
Effects of ultra-low-temperature cryopreservation on enzyme activity of silver pomfret sperm. (**A**) ATP content. (**B**) GR—glutathione reductase. (**C**) LDH—lactate dehydrogenase. Bars represent mean ± SD; different letters indicate significant differences.

The ATP content in fresh sperm was 1283.05 µmol/g protein, with LDH activity at 45.44 U/g and GR activity at 40.24 U/g. Cryopreserved sperm exhibited significantly reduced ATP content (644.94 µmol/g protein) compared to fresh sperm (*p* < 0.05). Both LDH and GR activities in cryopreserved sperm measured 37.43 U/g and 66.22 U/g, respectively, showing no significant difference from fresh sperm (Figure 6).

### 3.7. Effect of Ultra-Low-Temperature Cryopreservation on the Ultrastructure of Silver Pomfret Sperm

**Figure 7:**
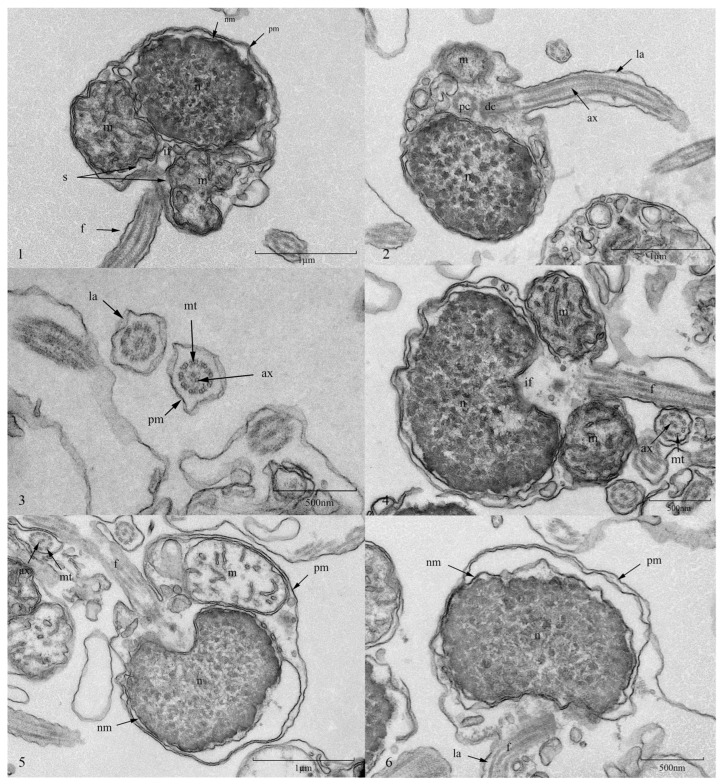
Ultrastructural changes in silver pomfret sperm before and after cryopreservation by TEM. (**1**–**3**) TEM micrographs of fresh sperm ultrastructure. (**4**) Cryopreserved sperm maintaining relative structural integrity. (**5**) Cryopreserved sperm demonstrating mitochondrial swelling, plasma membrane shrinkage in the head region, and enlarged perinuclear space. (**6**) Cryopreserved sperm exhibiting mitochondrial loss, cytoplasmic depletion, and flagellar detachment. (Abbreviations: pm. Plasma membrane; n. Nucleus; nm. Nuclear envelope; m. Mitochondrion; if. Implantation fossa; s. Sleeve cavity; pc. Proximal centriole; dc. Distal centriole (Basal body); ax. Axoneme; mt. Microtubule; v. Vesicle; f. Flagellum; la. Lateral fin).

TEM was employed to examine the ultrastructure of silver pomfret sperm before and after cryopreservation. Results revealed that the sperm head primarily consisted of an ovoid nucleus with densely aggregated chromatin. Both the nuclear envelope and plasma membrane exhibited irregularly undulating surfaces. The implantation fossa, located at the nuclear posterior, connected to the midpiece and contained sparse cytoplasm housing the centriolar complex. This complex comprised two components: a proximal centriole composed of nine triplet microtubules situated anteriorly within the implantation fossa, and a distal centriole (basal body) oriented perpendicularly to the proximal centriole, serving as the origin of the tail axoneme. Within the midpiece, two distinct structures were clearly observed: mitochondria and an asymmetrical sleeve surrounding the axoneme to form a sleeve cavity. Mitochondria within this cavity displayed a circular arrangement, featuring typical double-membrane morphology with distinct cristae projections and a relatively low electron-dense matrix. The tail flagellum, composed of the plasma membrane and axoneme, exhibited the characteristic “9 + 2” microtubule configuration: nine peripheral microtubule doublets encircling a central pair of singlet microtubules. Discontinuous undulating lateral fins extended bilaterally from the flagellar membrane. Transverse sections confirmed the near-circular contour of the tail, revealing no significant structures beyond the axoneme. Following cryopreservation, ultrastructural damage was observed in some sperm, primarily manifesting as plasma membrane shrinkage and rupture; enlarged perinuclear space; cytoplasmic loss; disorganized organelle distribution; mitochondrial swelling and structural disintegration; and midpiece–flagellum junction fractures. Notably, the fundamental microtubule architecture remained relatively intact (Figure 7).

## 4. Discussion

### 4.1. Determination of the Optimal Conditions for Cryopreservation of Silver Pomfret Sperm

#### 4.1.1. Appropriate Diluent: MPRS

Diluents create a suitable survival environment for sperm by adjusting parameters such as osmotic pressure, ionic composition, and pH [24]. This maintains normal physiological function and reduces the sperm’s metabolic energy expenditure [25]. Currently, no standardized diluent formula exists universally for fish sperm [26], necessitating selection based on the specific physiological characteristics of each species. Commonly used diluents include Hank’s, HBSS, MPRS, and Cortland, which differ in their regulation of osmotic pressure, maintenance of ionic balance, and energy provision, thereby directly influencing sperm preservation outcomes. In this study, MPRS, used as the diluent, yielded the optimal cryopreservation efficacy for silver pomfret sperm. This finding is consistent with reports in other marine teleosts, such as *Takifugu bimaculatus* [27], *Acanthopagrus latus* [28], *Epinephelus lanceolatus* [29], and *Lateolabrax japonicus* [30]. The motility, fast movement time, and movement time of sperm showed no significant difference from those of fresh sperm, indicating that MPRS provides a suitable microenvironment for silver pomfret sperm.

#### 4.1.2. Appropriate Cryoprotectant: EG

Sperm cryopreservation in marine fish is challenging due to the high susceptibility of sperm to freezing-induced structural damage [31,32]. While cryoprotectants such as EG, PG, DMSO, Gly, and MeOH [33] are widely used to mitigate ice crystal formation and stabilize cellular integrity, their efficacy is highly species-specific [34]. In this study, we systematically compared five common cryoprotectants for cryopreserving silver pomfret sperm and demonstrated that a protocol using 20% EG significantly outperforms others, as well as those commonly adopted in other marine fish species.

Notably, the optimized 20% EG condition achieved exceptional post-thaw motility parameters, underscoring its remarkable protective capacity. The superiority of EG is attributed to its low molecular weight and high membrane permeability, which facilitate rapid intracellular penetration, limit ice crystallization, and minimize mechanical injury to sperm structure [35]. In contrast, although DMSO at 10% has been successfully applied in red snapper (*Lutjanus argentimaculatus*) [36], it yielded suboptimal outcomes in silver pomfret, likely due to its higher cytotoxicity and tendency to provoke osmotic imbalance—factors that critically impair sperm survival and functional integrity [37].

#### 4.1.3. The Optimal Dilution Ratio: 1:6

The dilution ratio is a critical determinant of success in fish sperm cryopreservation, as it directly modulates the osmotic environment and the efficacy of cryoprotectant delivery. An optimal ratio ensures sufficient cryoprotectant permeation while avoiding toxicity, thereby maximizing post-thaw motility [38]. Given significant interspecific differences in sperm density and physiology, identifying a species-specific dilution ratio is essential.

In this study, we determined that a 1:6 dilution ratio was optimal for silver pomfret sperm, resulting in post-thaw motility parameters that significantly outperformed all other ratios tested. This optimal ratio is consistent with that reported for gilthead sea bream (*Sparus aurata*) [39], yet it provides a clearly defined and highly effective standard for a species of high aquaculture value where such protocols were previously unrefined.

The superiority of the 1:6 ratio can be attributed to two key factors: it ensures a uniform sperm distribution for consistent cryoprotectant interaction, and it likely maintains an adequate energy substrate concentration, thereby preserving sperm vitality throughout the freezing–thawing cycle. Our findings establish this dilution protocol as a superior and reliable standard for silver pomfret sperm cryopreservation, offering a tailored solution that enhances post-thaw quality beyond generic or suboptimal ratios.

#### 4.1.4. Optimal Cooling Height: 7 cm

Achieving an optimal cooling rate is critical to avoiding both intracellular ice formation during rapid cooling and prolonged osmotic stress during slow freezing [40]. The two-step cooling method, which regulates the cooling rate by varying the height above liquid nitrogen, is a standard yet highly tunable approach in fish sperm cryopreservation [41].

In this study, we systematically optimized this key parameter for silver pomfret sperm. Our results establish that a cooling height of 7 cm provides the ideal thermal profile, yielding exceptional post-thaw recovery. Sperm frozen at this height exhibited remarkable performance, motility, fast movement time, and movement time close to fresh sperm. This level of functional preservation is superior to the outcomes at other heights and aligns with the optimal condition reported for *Varicorhinus barbatulus* [42].

The 7 cm protocol represents a finely balanced cooling rate that effectively mitigates both ice-crystal damage and osmotic injury [43]. This finding provides a validated, high-efficacy standard for silver pomfret, underscoring the necessity of species-specific protocol optimization and highlighting the superior cryopreservation outcome achievable with our tailored approach.

#### 4.1.5. Optimal Thawing Temperature: 28 °C

Temperature is a critical factor influencing sperm motility and function, and the effectiveness of sperm cryopreservation is highly dependent on the thawing temperature. During sperm thawing, the initial melting of extracellular ice crystals causes an osmotic imbalance, leading to rapid water influx into the cells. This results in a sharp increase in cell volume; when the expansion exceeds the tolerance limit of the cell membrane, swelling-induced rupture occurs [20,44]. To minimize physical damage from ice crystals, prevent injury from solution concentration changes, and maximize sperm protection, thawing must rapidly traverse the temperature range most susceptible to damage. Common thawing methods include water bath thawing or thawing under running water at room temperature [45]. In this study, thawing at 28 °C yielded the best results for sperm, consistent with findings for Brazilian migratory fish [46]. Post-thaw sperm motility, fast movement time, movement time, and lifetime showed no significant difference compared to fresh sperm, demonstrating that a thawing temperature of 28 °C effectively maintains sperm motility and functional capacity, allowing thawed sperm to closely approximate the physiological state of fresh sperm.

### 4.2. Effect of Cryopreservation on Enzyme Activities in Silver Pomfret Sperm

In the fields of fish reproduction and germplasm resource preservation, sperm cryopreservation technology is critically important, with enzyme activity and ATP content serving as key indicators for assessing the quality of cryopreserved sperm. ATP plays a central role in physiological processes such as sperm motility and metabolism. Fluctuations in its concentration affect multiple cellular functions, and ATP levels exhibit a significant decline during apoptosis or necrosis [47]. In this study, the ATP content of cryopreserved sperm was significantly lower than that of fresh sperm, consistent with findings in cryopreservation studies of basa catfish (*Pangasius bocourti*) [48] and Atlantic salmon (*Salmo salar*) [47]. This decrease may be attributed to swelling and damage in sperm mitochondria following ultra-low-temperature treatment, impairing ATP synthesis [49]. Furthermore, the observed reduction in LDH enzyme activity in this study indicates disruption of energy metabolism pathways, indirectly reducing ATP production. Sperm motility requires substantial energy expenditure; insufficient ATP supply restricts sperm movement, hindering their ability to reach and fertilize eggs [50]. The reduction in ATP content implies diminished available energy for sperm upon revival, directly impacting their motility.

LDH provides essential energy support for sperm motility by participating in energy metabolism processes and is closely associated with sperm motility performance and energy supply efficiency [51]. In silver pomfret, LDH enzyme activity was lower in cryopreserved sperm compared to fresh sperm (*p* > 0.05), consistent with findings from cryopreservation studies on other fish species such as viviparous black rockfish (*Sebastes schlegelii*) [52] and black seabream (*Acanthopagrus schlegelii*) [15]. The reduced LDH activity indicates suppression of sperm energy metabolism pathways and weakened glycolysis under anaerobic conditions, compromising the energy supply to sperm [53]. This impairment is likely attributable to structural damage to the LDH enzyme caused by the freezing process, which disrupts its normal catalytic function [15].

GR effectively scavenges excess intracellular ROS, thereby protecting the integrity of the cell membrane structure [54]. In this study, GR enzyme activity increased in cryopreserved–thawed silver pomfret sperm, consistent with findings from cryopreservation studies on Russian sturgeon (*Acipenser gueldenstaedtii*) sperm [54]. The elevated GR activity indicates that sperm undergo oxidative stress during the freezing process, leading to excessive ROS generation within the cells. Consequently, the organism’s antioxidant system enhances GR activity to bolster antioxidant defense capacity and mitigate the impact of oxidative damage on sperm.

### 4.3. Effect of Cryopreservation on the Ultrastructure of Silver Pomfret Sperm

The ultrastructure of sperm serves as a crucial basis for evaluating sperm quality before and after cryopreservation [22]. During ultra-low-temperature cryopreservation, abrupt temperature changes can trigger abnormal intracellular ice crystal formation, causing systematic damage to the sperm ultrastructure and impairing sperm motility [55]. Following cryopreservation, alterations were observed in the morphology and internal structure of silver pomfret sperm. TEM observations further confirmed cryo-induced damage to the internal sperm structure. Some sperm exhibited ruptured or missing plasma membranes, and an enlarged space between the nuclear membrane and plasma membrane. Cytoplasmic loss and abnormal organelle distribution were also observed. Mitochondria displayed characteristic pathological alterations, including swelling of the matrix region and fragmentation of cristae. While microtubule structures remained largely intact, the detachment and fracture of flagella indicated a significant adverse impact of the freezing process on sperm motility function. Compared to cryodamage observed in sperm from other teleost fish, rainbow trout (*Oncorhynchus mykiss*) sperm exhibited typical post-cryopreservation damage features such as compromised plasma membrane integrity, abnormal mitochondrial swelling, and flagellar breakage [56]. Similarly, cryopreserved sperm from Pabdah catfish (*Ompok pabda*) showed significantly reduced fertilization success rates and hatching rates compared to fresh sperm [57]. These findings collectively demonstrate that the mechanisms of cryodamage to fish sperm during the freezing process are universal. However, an assessment of sperm fertility was not performed and we highlight this as a key limitation of the study.

## 5. Conclusions

Through evaluation diluent types, cryoprotectant types and concentrations, dilution ratios, cooling height, and thawing temperature, the optimal protocol for ultra-low-temperature cryopreservation of silver pomfret sperm was determined as follows: MPRS extender supplemented with 20% EG cryoprotectant, diluted at a ratio of 1:6 (*v*/*v*), exposed to liquid nitrogen vapor at 7 cm above the liquid nitrogen surface for 5 min before plunging into liquid nitrogen, and thawed in a 28 °C water bath for 10 s. This protocol yielded post-thaw sperm motility exceeding 90%, meeting the standards for practical application. Given the constraints of time and experimental conditions, this study focused solely on the techniques and ultrastructural aspects of low-temperature and ultra-low-temperature cryopreservation for silver pomfret sperm. Further in-depth research is warranted on the fertilizing capacity of cryopreserved sperm, cryogenic injury, and alterations in genetic material.

## Figures and Tables

**Table 1:** Composition of the tested extenders (prepared in distilled water).

Component (mM)	HBSS	MPRS	Cortland	Ringer’s	Hank’s
NaCl	136.89	60.40	124.06	111.23	136.89
KCl	5.37	5.23	5.10	1.88	5.37
CaCl_2_	-	-	1.62	-	1.26
CaC1_2_·2H_2_O	1.09	1.16	-	0.82	-
NaHCO_3_	4.17	2.98	11.9	2.38	4.17
KH_2_PO_4_	0.44	-	-	-	0.44
MgSO_4_·7H_2_O	0.81	-	0.93	-	0.41
MgCl_2_·6H_2_O	-	1.13	-	-	0.49
Na_2_HPO_4_·7H_2_O	0.45	-	-	-	-
NaH_2_PO_4_	-	1.83	-	0.08	-
NaH_2_PO_4_·2H_2_O	-	-	2.63	-	-
Na_2_HPO_4_·12H_2_O	-	-	-	-	0.18
Glucose	5.55	55.51	5.55	-	55.51
pH	7.2	6.68	7	7.4	6.8

## Data Availability

Data will be made available on request.
